# Microbial succession from nursery to vineyard highlights the role of beneficial and pathogenic microbes in young vineyard yield

**DOI:** 10.1186/s40793-026-00905-8

**Published:** 2026-05-04

**Authors:** Colin Todd, Philippe E. Rolshausen

**Affiliations:** University of California, Riverside, 92521 USA

**Keywords:** Pathobiome, Young vine decline, Esca, Black foot, Terroir, Machine learning, Predictive modeling

## Abstract

**Background:**

The grapevine microbiome plays a central role in shaping vineyard performance, yet the influence of nursery inherited microbes on vineyard establishment and early productivity remains poorly understood. Our goals were to study the endosphere and rhizosphere microbiome succession as grapevine transition from nursery to vineyard and determine in what capacity the endogenous microbiome from the nursery shapes vineyard outcomes.

**Results:**

We profiled, using amplicon-based sequencing, the fungal and bacterial communities across five bio-compartments (scion graft union, rootstock graft union, crown, roots, and rhizosphere) from two sets of grafted vines (Cabernet Sauvignon and Chardonnay grafted on 1103P rootstock) originating from two nurseries and followed their succession over three years after planting in a commercial vineyard. Nurseries produced vines with distinct endospheric microbiomes that converged overtime but that remained significantly different after three years. Microbial turnover occurred at a much faster pace in belowground (root and rhizosphere) compared to trunk compartments post-planting, with 15% of the initial microbes persisting in three-year-old vineyard. The fungal pathobiome partially inherited from nurseries and associated with vascular diseases of the trunk and root was also clearly distinct after three years. Yet, we did not observe typical disease symptoms development or vine death as we would expect, likely because vines were not under stress during the experimental timeframe. Vineyard yield was highly variable among clonal vines, and statistical modeling revealed that a narrow subset of amplicon sequence variants (ASVs) explained a large portion of this variance. Regression models using the top ten high-impact ASVs accounted for 51% and 60% of yield variation in trunk and belowground compartments, respectively. Notably, 16 of the 19 yield-associated ASVs originated from nurseries, underscoring the long-term influence of nursery-derived microbes on vineyard success.

**Conclusion:**

These findings highlight the dual role of beneficial and pathogenic nursery microbiota in shaping grapevine performance. It also suggests that the nursery life stage could be leveraged to engineer the grapevine microbiome and improve vineyard resilience.

**Supplementary Information:**

The online version contains supplementary material available at 10.1186/s40793-026-00905-8.

## Introduction

Grapevine is a significant crop globally, spanning approximately 7 million planted hectares, valued at over 30 billion dollars, and is vital to the economy of many regions [1, 2]. Rising consumer demand for sustainably produced crops and stricter regulations on synthetic agrochemicals have created a booming market for novel biopesticides, biostimulants, and biofertilizers. As a result, the grapevine microbiome has become an active area of research for developing biologically-based agronomic tools that can enhance disease management, improve resistance to abiotic stress, and increase crop yield along with fruit quality [3, 4].

The microbiome has been identified as an integral part of the grape ‘terroir’, a term used to describe the identity of a viticulture region. Factors that shape this identity are multiple and include among others biogeography, climate conditions, soil characteristics, farming practices, rootstock and scion grape cultivars [5–8]. There is now clear evidence that defined viticulture areas have distinct microbial signatures that contribute to wine organoleptic properties [5, 9, 10], and that soil serves as a reservoir of autochthonous microbes [10, 11]. Dispersion of soilborne microbes on aboveground tissues is thought to occur through airborne dust particles, via water splash, or insect vectors [12]. Internally, movement of endophytic microbes by way of the plant sap from the belowground to aboveground organs has also been shown [13]. Microbial communities are partitioned spatially in the various host organs and bio-compartments [10, 11, 13]. Additionally, the microbial communities are temporally structured with habitat patterns according to the development stage of grapevines [11, 14].

While the origin of epiphytic microbes and their impact on plant biological processes have been well studied, those living inside grapevine (endophytic) are less well understood. Grapevine inherits microbes from the nursery and some questions about the longevity of this native microbiome and its long-term impact on vineyard productivity remain unanswered [13, 15]. Presence of fungal and bacterial pathogens affecting the host root and vascular system of grapevine have been clearly linked to nurseries and their spread to propagation practices [15]. Once vines are planted in the vineyard, these pathogens can cause diseases known as young vine decline (a.k.a. young esca), black foot and crown gall although abiotic stresses appear to be a key determinant in disease outcome [16]. Fungal species complexes have been associated with young vine decline (*Phaeoacremonium minimum*, *Phaeomoniella chlamydospora*, *Fusarium* species) and black foot (*Dactylonectria*, *Ilyonectria*, *Campylocarpon*). Biological control agents such as *Trichoderma* are also commonly identified in plant nursery stocks and have shown to provide protection against grapevine trunk and root pathogens [17, 18]. However, the presence of naturally occurring plant growth promoters or mutualistic organisms associated with nursery vines and their impact on vine productivity in young vineyard post-planting has remained unanswered.

The research goals were to study the microbiome dynamic from nursery to vineyard and determine in what capacity microbial inheritance from nursery shapes the vineyard microbiome. We hypothesize that the microbial communities inhabiting the grapevine endosphere are relatively stable and shaped by nursery mother vines and cultural practices. In contrast, the communities composing the rhizosphere and root endosphere are dynamic and largely influenced by local vineyard soil community composition. We also tested the hypothesis that the grapevine microbiome contributed meaningfully to crop yield and determined if the native microbiome from the nursery contributed to this effect. The outcome from this work underscores the importance of the nursery life stage on the microbiome legacy and its implication on the scientific basis of grapevine terroir.

## Materials and methods

### Experimental design and sample collection

Chardonnay clone 04 and Cabernet Sauvignon clone 08 grafted on 1103P rootstock were collected from two California nurseries in 2020. A total of eighty vines were initially processed at time zero before planting for endophytic microbiome profiling from five different bio-compartments: the scion graft union, the rootstock graft union, the crown, the roots and the rhizosphere. An additional 160 vines were planted at a density of 2.3 m (vine spacing) x 3.3 m (row spacing) in the spring of 2020 in a commercial vineyard near Madera, California. The experimental design consisted of twenty random blocks of 8 vines, with each block representing one cultivar and one nursery and replicated five times in the vineyard. Vines were trained in a VSP (Vertical Shoot Positioning) system with the two bilateral cordons at 1.5 m above the soil line. Standard viticulture practices (e.g., irrigation, pruning, fertilizer and pesticide application) were implemented uniformly in the vineyard for three consecutive years after planting. In 2021 and 2022 one vine from each block was randomly collected and brought back to the lab to profile the microbiome from all five bio-compartments. Additionally, the lowest and highest yielding vines from each block were collected in 2023 and brought back to the lab to be processed in the same way.

### Phenotypic measurements

Trunk diameter was measured in 2021, 2022, and 2023 using an electronic caliper. Two perpendicular measurements were taken at the midpoint of each vine trunk and averaged to obtain the diameter. Cane weight was measured after completely pruning each individual vine after harvest and weighing the resulting plant material using an electronic scale. Vines were harvested at three years post-planting in September of 2023 since it is considered to be the first fully productive year. Fruit yield was measured for every single vine by removing all the fruit and weighing it on an electronic scale.

### Sample processing for total DNA extraction

Samples were processed utilizing previously established methods [13]. The rhizosphere was collected by submerging freshly collected roots in a sterile 50 mL Falcon tube containing 25 mL PBS with 0.02% Silwet L-77 and vortexing at maximum speed for 15 s. The cleaned roots were then placed into new sterile containers for further processing. The dirty tubes were centrifuged for 15 min at 3200 g to allow for the fine sediment to form a pellet at the bottom of the tube. The supernatant was discarded leaving only the rhizosphere. Wood tissue pieces from the four bio-compartments (root, crown, above and below the graft union) were washed in sterile water and surface sterilized with ethanol before being flamed on a Bunsen Burner. Samples were then lyophilized using the Labconco FreeZone benchtop freezer dryer for three days to remove any water from the samples. Dried samples were completely ground to powder using the Retsch MM400 mill grinder. Total DNA from 100 mg of each sample type was extracted from each bio-compartment using the ZymoBIOMICS DNA Miniprep Kit.

### Microbiome library preparation and sequencing

Extracted DNA was used to amplify the ITS or 16S region with barcoded primers that are unique for each sample. The Earth Microbiome Primers were used for amplifying the ITS1 region (forward: CTTGGTCATTTAGAGGAAGTAA – reverse: GCTGCGTTCTTCATCGATGC), while slightly altered primers that exclude chloroplasts and mitochondria were used to amplify the v4 16S region (forward: GTGYCAGCMGCCGCGGTAA – reverse: ACCMGGGTATCTAATCCKGT) [19]. The protocol for creating the ITS amplicons is as follows: 10 µL of 2x Apex Hot Start Master Mix, 0.5 µL of Forward Primer 10 µM, 0.5 µL of Reverse Barcoded Primer 10 µM, 2 µL of template DNA, and 12 µL of H20 for a final volume of 25 µL. The PCR conditions were 95 °C for 15 min, 35 cycles of 95 °C for 30 s, 62 °C for 30 s, 72 °C for 1 min, followed by 72 °C for 5 min. The protocol for creating the 16S amplicons is as follows: 10 µL of 2x Apex Hot Start Master Mix, 0.5 µL of Forward Barcoded Primer 10 µM, 0.5 µL of Reverse Primer 10 µM, 0.5 µL of mitochondrial PNA clamps 5 µM, 0.5 µL of plastid PNA clamps 5 µM, 2 µL of template DNA, and 11 µL of H20 for a final volume of 25 µL. The PCR conditions were 95 °C for 15 min, 35 cycles of 95 °C for 30 s, 66 °C for 30 s, 72 °C for 1 min, followed by 72 °C for 5 min. Each PCR run included a negative control with no template DNA to ensure that the sequenced reaction products were directly derived from samples. Each PCR product was run on an agarose gel using electrophoresis to ensure the correct amplification occurred and pooled into approximately equimolar libraries using a previously established protocol [20]. The pooled libraries were then sequenced using the Illumina Miseq 2 × 300.

### Computational analysis

Sequencing reads were demultiplexed according to their barcodes and sorted into the appropriate sample bucket. A quality check was performed on the reads using fastqc in Bash. Based on the sequencing quality report, the paired-end reads were trimmed using trimmomatic with the parameters LEADING:23, TRAILING:23, SLIDINGWINDOW:7:25, and MINLEN:50 for both the ITS and 16S sequences. Paired-end reads were then merged using pear in Bash before being imported into R for the remaining processing using the DADA2 [21] and phyloseq [22] libraries. Reads were further filtered to remove sequences containing ambiguous nucleotides (Ns) using the filterAndTrim function and dereplicated using the derepFastq function. The error rates for the sequences were learned with the learnErrors function and used by the dada function to identify true amplicon sequence variants (ASVs) and further filter out sequence variants produced by sequencing errors. A final sequence table was produced by combining all inferred ASVs using the makeSequenceTable function and removing chimeras using the removeBimeraDenovo function (Supplemental Tables 1 and 2). Each sequence was then given a taxonomic assignment with the assignTaxonomy function and a minimum bootstrap confidence of 50 utilizing the UNITE version 4.4.2024 database for ITS and the Silva SSU v138.1 database for 16S. The sequence tables, taxonomy assignment tables, and metadata mapping files were then combined using the phyloseq library to create two phyloseq objects for the ITS and 16S sequences. The phyloseq objects were then filtered to remove any ASVs that were unable to be assigned at the Phyla level and any ASVs that had fewer than 10 reads. Both the ITS and 16S phyloseq objects were then rarefied to a depth of 10,000 reads each according to a rarefaction analysis that suggested such a depth adequately balanced ensuring sequencing of rare reads while also retaining over 80% of the total samples (Supplemental Fig. 1). The rarefied phyloseq objects were then combined based on matching Sample ID and used for downstream analyses requiring both datasets.

**Table 1:** Percent incidence and abundance of fungal pathogens associated with grapevine trunk diseases in nurseries (year 0) and in three-year-old vineyard

Disease	Taxa	Nursery 2020		Vineyard 2023	
		Percent Incidence	Percent Abundance	Percent Incidence	Percent Abundance
Young Vine	Phaeoacremonium	51.1	3.1	65.4	11.1
	Phaeomoniella	3.7	< 0.1	4.5	0.8
	Pleurostoma	13.9	0.4	2.3	0.1
	Cadophora	2.2	< 0.1	0.8	< 0.1
	Fusarium	100	12.9	95.5	6.2
Black Foot	Dactylonectria	42.3	2.5	47.4	9.5
	Cylindrocladiella	11.7	< 0.1	24.1	0.5
	Rhizoctonia	2.2	< 0.1	24.1	0.5
	Ilyonectria	0	0	6.8	0.1
	Truncatella	0	0	9	< 0.1
	Neonectria	16.1	0.2	1.5	0.3
	Campylocarpon	3.7	< 0.1	0.8	0.02
Botryosphaeria	Diplodia	2.9	< 0.1	0	0
Canker	Neofusicoccum	2.2	< 0.1	0.8	< 0.1
Phomopsis	Diaporthe	2.9	< 0.1	0	0

ASVs explain a significant portion of yield variance

Differential abundance analyses were conducted using ANCOMBC2 [23] on microbial communities from trunk- and rhizo-compartments samples collected in 2023. Relative abundances were calculated for each ASV, and analyses were performed with vigor (“Low” vs. “High”) as the fixed effect. Significant taxa were identified based on adjusted p-values (*P* < 0.05). Indicator species analysis [24] was performed using the indicspecies package to identify ASVs significantly associated with vigor in both trunk and belowground datasets, applying a false discovery rate correction. LASSO regression was implemented using the glmnet package [25]. Spearman correlation was also used to identify ASVs significantly correlated with yield (*P* < 0.05) [26]. Multicollinearity was assessed by computing correlation matrices for the selected ASVs, and highly correlated ASVs (with correlation coefficients exceeding 0.9) were identified and removed to enhance model stability. This reduction resulted in a refined set of ASVs for both trunk and belowground analyses.

Random Forest models were then constructed for both trunk and belowground datasets using the randomForest package, with 5,000 trees to assess variable importance [27]. The models included yield as the dependent variable and incorporated selected ASVs along with covariates such as cultivar and nursery origin. The mtry parameter was tuned using the tuneRF function to optimize model performance. Variable importance was determined by percent increase in the mean squared error and for both trunk and belowground samples the top 10 ASVs were identified. Multiple linear regression models were developed using the top ASVs identified from the Random Forest analyses. Separate models were fitted for trunk and rhizo-compartment samples using the lm function, incorporating significant ASVs and cultivar information as predictors. To validate the robustness of these regression models, we employed 10-fold cross-validation using the caret package, partitioning the data into 80% training and 20% testing subsets. Performance metrics, including Root Mean Square Error (RMSE) and R-squared values, were calculated for both training and testing datasets to assess the models’ accuracy and generalizability.

## Results

### Vines from different nurseries display distinct microbiomes that converge following planting

**Fig. 1:**
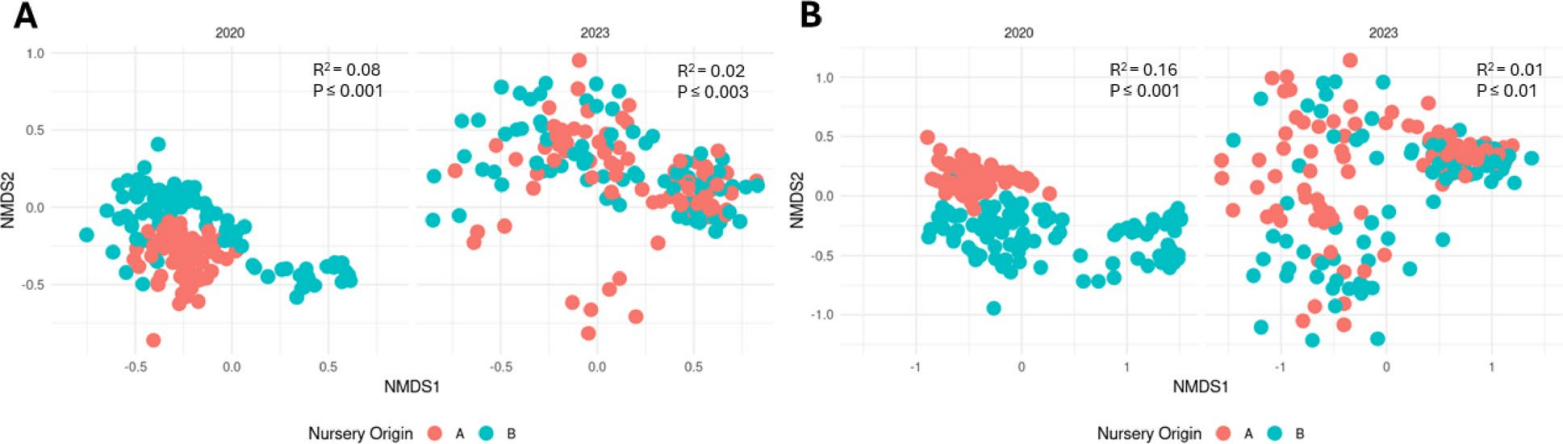
Beta diversity of the fungal (**A**) and bacterial (**B**) microbiome in grapevine depicted by NMDS (Non-Metric Multidimensional Scaling) ordination plots based on Bray-Curtis dissimilarity in the nurseries (2020) and the final year in the vineyard (2023). PERMANOVA (Permutational Multivariate Analysis of Variance) was used to test for significant differences between groups within each graph. The R2 (explained variance) values indicate the percentage of the variation in the dissimilarity matrix that can be attributed to the grouping variable. (**A**) Fungal microbiome beta diversity grouped by different nurseries in 2020 (*P* < 0.001; R^2^ = 0.08) and 2023 (*P* < 0.01; R^2^ = 0.02). (**B**) Bacterial microbiome beta diversity grouped by different nurseries in 2020 (*P* < 0.001; R^2^ = 0.16) and 2023 (*P* < 0.01; R^2^ = 0.01)

The plant materials from the two nurseries were of identical genetic backgrounds, yet their microbiome composition differed significantly. Non-Metric Multidimensional Scaling (NMDS) ordination plots based on Bray-Curtis dissimilarity analysis showed significant differences in the microbial composition at time 0 for both the fungal (*P* < 0.001 PERMANOVA; Fig. 1A) and bacterial (*P* < 0.001 PERMANOVA; Fig. 1B) microbiomes, with nursery origin explaining 8% and 16% of the microbial dissimilarity, respectively. The fungal and bacterial richness of nursery vines at time 0 was comparable for both cultivars (Wilcoxon signed-rank test; Supplemental Fig. 2A and B). Although the Bray-Curtis dissimilarity was still significant for the mycobiome (*P* < 0.01 PERMANOVA; Fig. 1A) and bacteriome (*P* < 0.01 PERMANOVA; Fig. 1B) composition at 3 years post-planting, nursery origin explained only 2% and 1% of their microbiome dissimilarities respectively.

### The grapevine microbiome assemblage is affected spatially and temporally

**Fig. 2:**
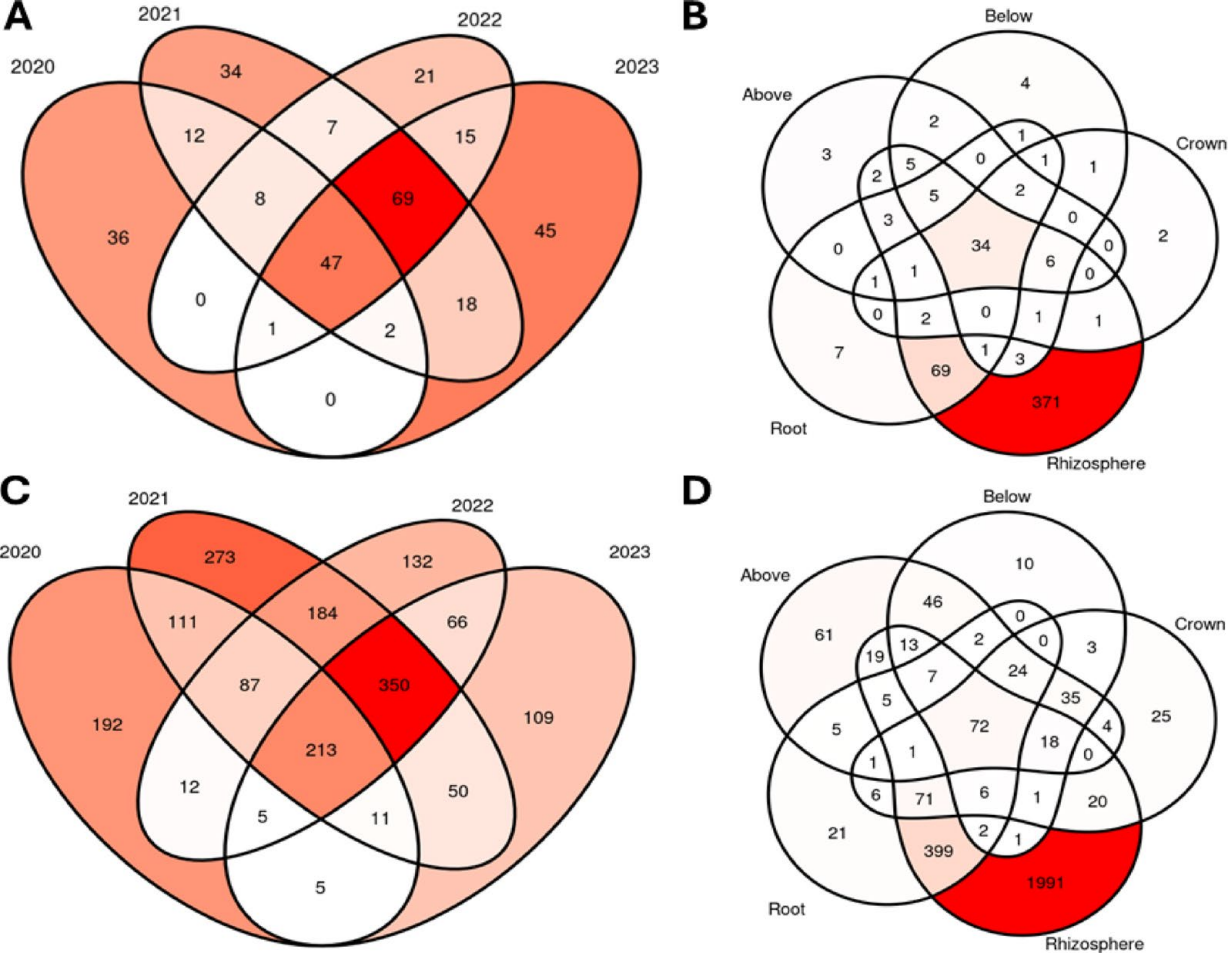
Venn diagrams illustrating the presence or absence of microbial taxa (ASVs) across time and space. The Venn diagrams show the distribution of fungal taxa across the different years the samples were collected (A) and bio-compartments (B). Similarly, the second set of Venn diagrams show the distribution of bacterial taxa across the different years the samples were collected (C) and bio-compartments (D). Taxa must be present in at least 10% of samples within each group to be considered

The microbial taxa present during the nursery to vineyard transition varied through both time and space (Fig. 2). Although many distinct taxa were only present in certain years or bio-compartments, there were also a core set of fungi and bacteria that were observed in all four years (15% and 12% of the total ASVs respectively) and all five bio-compartments (6.4% and 2.5% of the total ASVs respectively). The grapevine mycobiome richness slightly increased overtime but was not statistically significant (*P* > 0.05 Kruskal-Wallis; Supplemental Fig. 3A), whereas there was a stark increase in bacterial richness from year 0 to year 1 that decreased in subsequent years (Supplemental Fig. 3B). Venn diagrams showed distinct microbial communities each year (Fig. 2A and C), but the highest number of ASVs were measured in year 1 to 3 for both fungi (22% of all ASVs) and bacteria (19% of all ASVs). The five bio-compartments also harbored distinct sets of both fungi (Fig. 2B) and bacteria (Fig. 2D), but the rhizosphere was the compartment that supported the majority of unique taxa identified (70% for both fungi and bacteria). Hence, the rhizosphere displayed higher taxonomic richness than the vine endosphere, and the root showing the most fungal and bacterial biodiversity of all the endospheric compartments (*P* < 0.001 Kruskal-Wallis; Supplemental Fig. 3C and D).

**Fig. 3:**
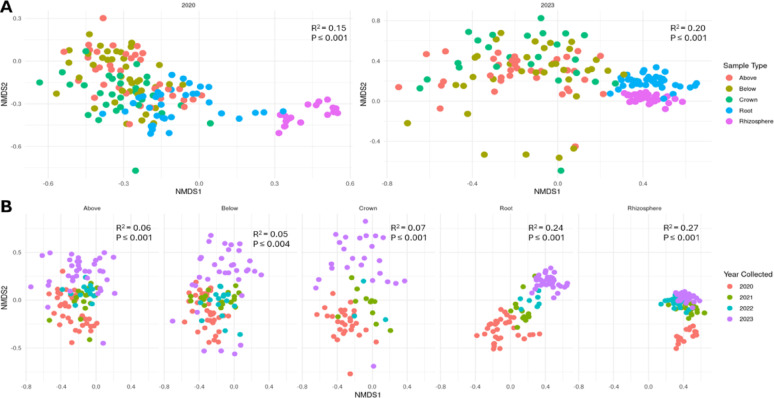
Beta diversity of the fungal microbiome in grapevine depicted by NMDS (Non-Metric. Multidimensional Scaling) ordination plots based on Bray-Curtis dissimilarity. PERMANOVA (Permutational Multivariate Analysis of Variance) was used to test for significant differences between groups within each dataset. The R2 (explained variance) values indicate the percentage of the variation in the dissimilarity matrix that can be attributed to the grouping variable. (A) Fungal microbiome beta diversity grouped by different bio-compartments (above the graft union, below the graft union, crown, root, and rhizosphere) in 2020 nursery vines (*P* < 0.001; R^2^ = 0.15) and 2023 field vines (*P* < 0.001; R^2^ = 0.2). The fungal community in the trunk (crown, above and below the graft union) and rhizo-compartments (root and rhizosphere) were statistically different *(P* < 0.001) and explained 7% and 13% of the variance in the dataset in 2020 and 2023, respectively. (B) The fungal microbiomes for each individual bio-compartment were grouped by year collected

**Fig. 4:**
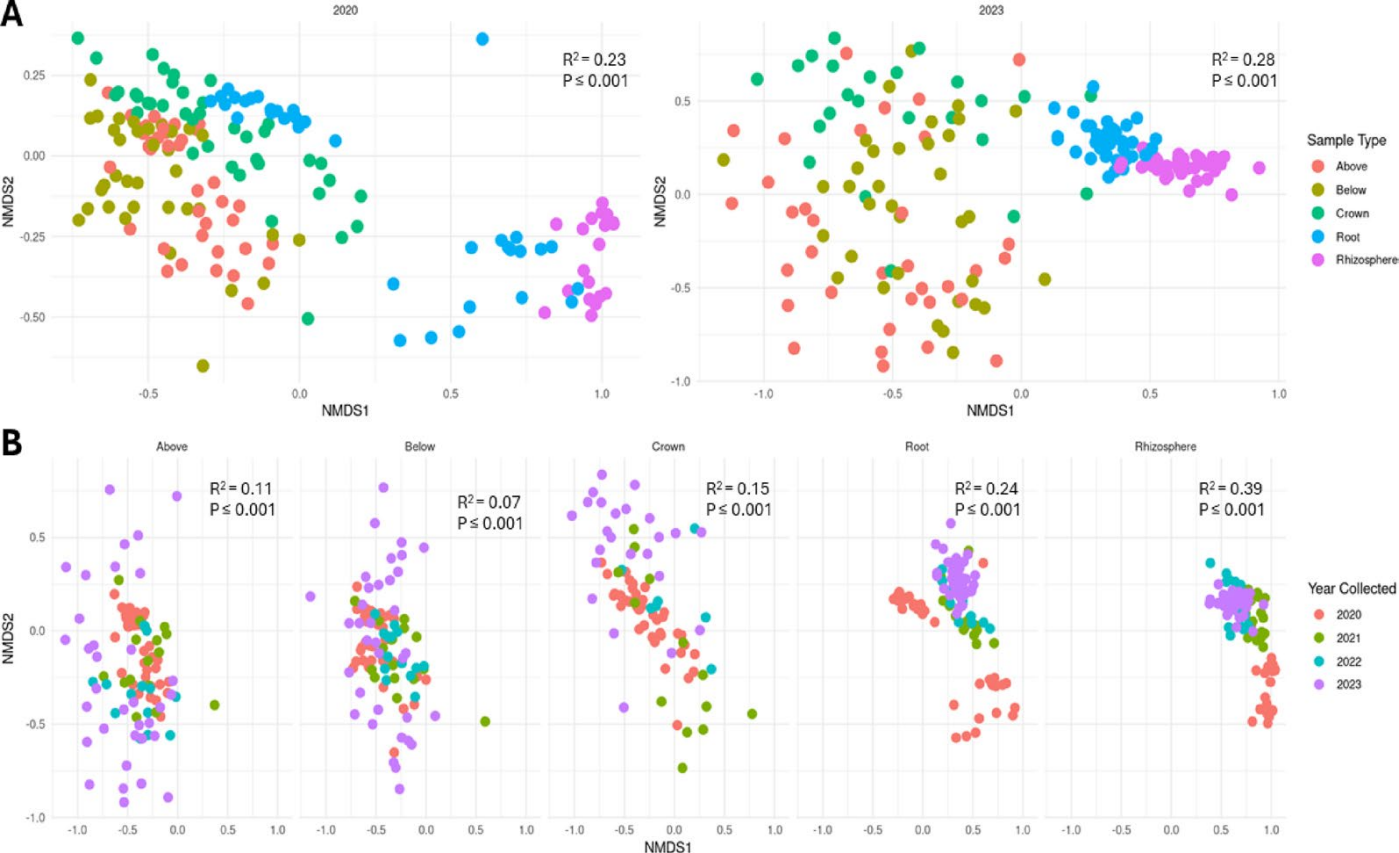
Beta diversity of the bacterial microbiome in grapevine depicted by NMDS (Non-Metric Multidimensional Scaling) ordination plots based on Bray-Curtis dissimilarity. PERMANOVA (Permutational Multivariate Analysis of Variance) was used to test for significant differences between groups within each graph. The R2 (explained variance) values indicate the percentage of the variation in the dissimilarity matrix that can be attributed to the grouping variable. (**A**) Bacterial microbiome beta diversity grouped by different sample type bio-compartments (above the graft union, below the graft union, crown, root, and rhizosphere) in 2020 nursery vines (*P* < 0.001; R^2^ = 0.23) and 2023 field vines (*P* < 0.001; R^2^ = 0.28). The fungal community in the trunk (crown, above and below the graft union) and rhizo-compartments (root and rhizosphere) were statistically different *(P* < 0.001) and explained 14% and 17% of the variance in the dataset in 2020 and 2023, respectively. (**B**) The bacterial microbiomes for each individual bio-compartment were grouped by year collected

NMDS ordination plots based on Bray-Curtis dissimilarity analysis indicated that the bio-compartment was a significant factor in shaping the fungal (*P* < 0.001 PERMANOVA; Fig. 3A) and bacterial (*P* < 0.001 PERMANOVA; Fig. 4A) microbiome, explaining 15% and 23% of the microbial variance in the 2020 nursery vines, respectively. In the 2023 grapevines, the bio-compartments accounted for an even higher amount of the microbial variance with 20% for the fungal and 28% for the bacterial datasets. Notably, there was a clear clustering shift of the root microbes with the rhizosphere over that period that accounted for the increased measure of variance (Figs. 3A and 4A). Plotting the Bray-Curtis dissimilarities using NMDS ordination plots supported that within each bio-compartment, the sampling year was a significant factor in the structure of their microbiomes, albeit with unequal contributions. The R-squared values indicated that the microbiomes in the trunk compartments (crown, above and below the graft union) were more stable over time in comparison to those of the rhizo-compartments (Figs. 3B and 4B). Additionally, the mycobiome generally was more stable over time when compared to the bacteriome, notably in the rhizosphere.

### The grapevine pathobiome shifts during the transition from nursery to vineyard

**Fig. 5:**
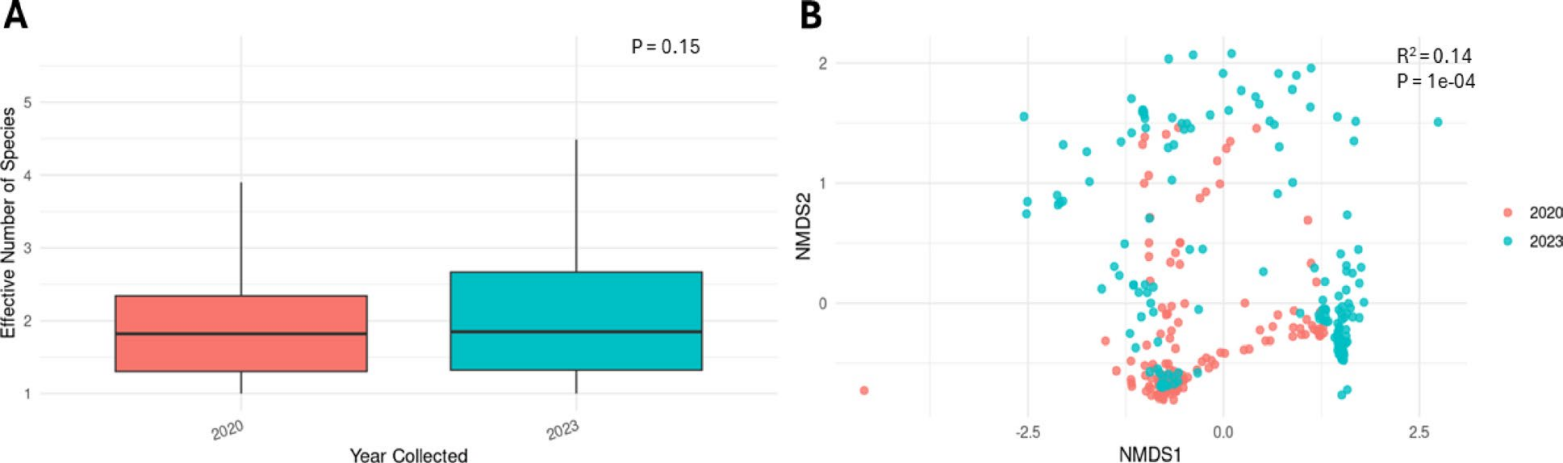
**A** Boxplot showing the effective number of pathogenic fungal taxa detected in grapevine tissues collected in 2020 and 2023 (Wilcoxon signed-rank test, *P* > 0.05), **B** non-metric multidimensional scaling (NMDS) ordination based on Bray–Curtis dissimilarities illustrate significant differences in pathogen community (pathobiome) composition between years. PERMANOVA (Permutational Multivariate Analysis of Variance) was used to test for significant differences between groups. The R2 (explained variance) values indicate the percentage of the variation in the dissimilarity matrix that can be attributed to the grouping variable. The pathobiome composition was significantly different between 2020 and 2023 (*P* < 0.001; *R*^*2*^ = 0.14)

A total of 10 (~ 12%; 6 Cabernet Sauvignon and 4 Chardonnay) and 3 (~ 4%; 1 Cabernet Sauvignon and 2 Chardonnay) vines were lost from each nursery in the first year following planting to unknown causes, but no vines died in subsequent years. The transition of grapevines from nursery to commercial vineyard settings significantly altered the community composition of fungal pathogens associated with grapevine trunk diseases. Although alpha diversity metrics did not indicate an increase in pathogen richness over time (*P* > 0.05 Wilcoxon signed-rank test; Fig. 5A), an NMDS ordination plots based on Bray-Curtis dissimilarity analysis showed distinct pathobiome profiles between 2020 and 2023 (*P* < 0.001 PERMANOVA; Fig. 5B).

Several latent pathogens that originated from the nurseries displayed a noticeable increase in both incidence and abundance from T0 to T3 (Table 1), including *Phaeoacremonium* and *Phaeomoniella*, the main pathogens linked to young vine decline, as well as *Dactylonectria*,* Cylindrocladiella* and *Rhizoctonia*, associated with black foot disease. On the other hand, several pathogens that contribute to young vine decline (*Fusarium*, *Pleurostoma* and *Cadophora*), black foot disease (*Neonectria*, *Campylocarpon*), bot canker (*Diplodia*, *Neofusicoccum*) and Phomopsis dieback (*Diaporthe*) decreased during that time frame. Finally, pathogenic fungi that were not present in the nursery vines were detected in three-year-old vines and included the black foot pathogens *Truncatella* and *Ilyonectria*.

**Fig. 6:**
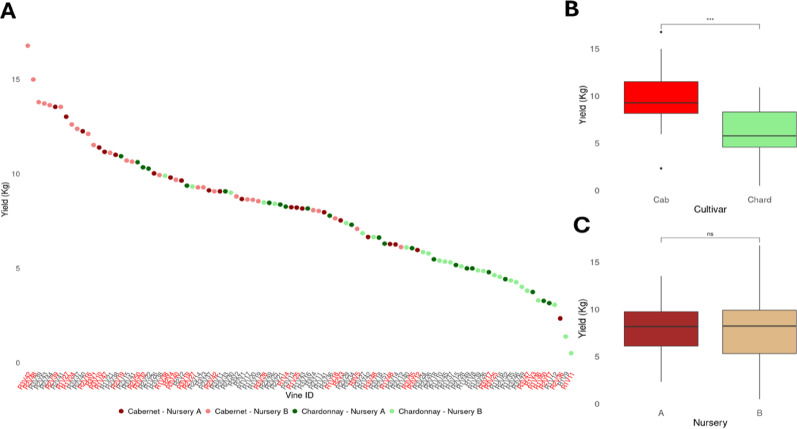
Plot shows yield data from the 100 vines remaining in the third year with each dot representing an individual vine and the color code corresponding to its cultivar and nursery of origin. (**A**) Red Vine ID labels on the X axis were the high or low yielding vines selected for further analysis. Wilcoxon signed-rank test showed yield difference between Cabernet Sauvignon and Chardonnay (*P* < 0.001; (**B**), but not between the two nurseries (*P >* 0.05; C)

A wide range in fruit yield was recorded at harvest in the last year of the experiment (2023) for each cultivar; Cabernet Sauvignon yield ranged from 2.3 kg to 16.8 kg, while Chardonnay ranged from 0.5 kg to 10.9 kg (Fig. 6A). A significant cultivar effect (*P* < 0.001 Wilcoxon signed-rank test; Fig. 6B) was found with the average yield of Cabernet Sauvignon (9.8 kg) and Chardonnay (6.2 kg). No significant difference in yield was found between vines from the two different nurseries (Fig. 6C). Our data also indicated that there was a strong correlation between yield in year 3 and pruning weight and trunk diameter for year 2 (R^2^ = 0.72 and 0.73, respectively) and year 3 (R^2^ = 0.67 and 0.65, respectively).

A total of 19 high and 19 low yielding vines were selected for from the replicated blocks. Amongst the high yielding vines the average yield was 12.7 kg for Cabernet Sauvignon and 9.3 kg for Chardonnay, while the low yielding vines produced 7.1 kg and 3.7 kg for Cabernet Sauvignon and Chardonnay, respectively. There were no significant differences in microbiome and pathobiome richness or composition (Supplemental Figs. 4 and 5) between high and low yielding vines suggesting that these factors were not drivers of the phenotypes.

**Fig. 7:**
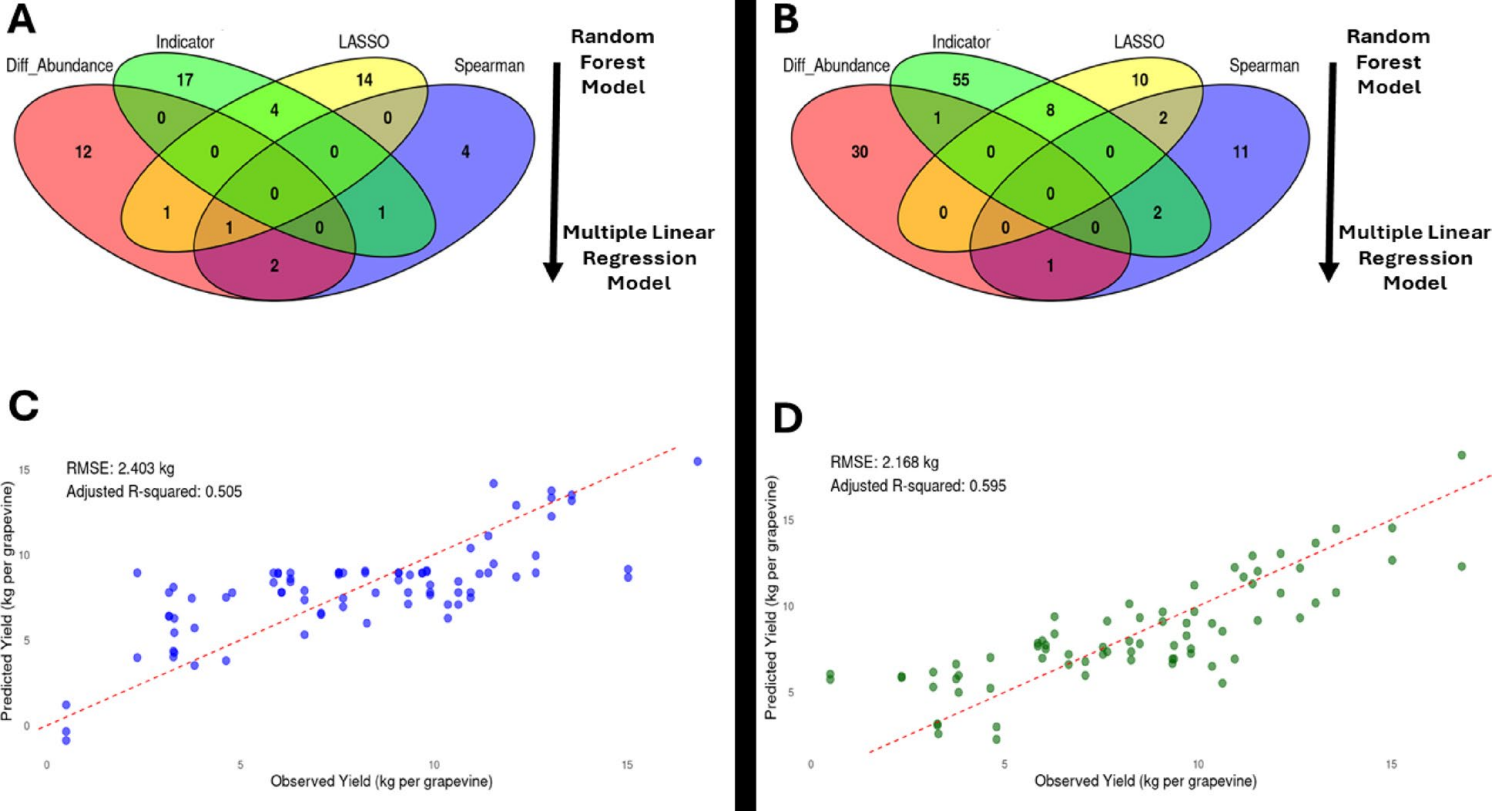
A combination of methods were used to identify ASVs that are associated with either high or low yielding vines. A random forest model was used to identify the top ten ASVs with the highest impact on yield. The field data was partitioned into trunk compartments (**A**) and rhizocompartments (**B**), and the top ten ASVs were identified for each using these distinct datasets. The multiple linear regression models using trunk (**C**) or rhizo- (**D**) compartments high impact ASVs explain 51% or 60% of total yield variance, while controlling for cultivar differences. The trunk regression model has a p-value of 2.7e-09 and the root regression model has a p-value of 1.017e-10

Four distinct statistical methods (Differential Abundance, Indicator Species, LASSO regression, and Spearman correlation) were used to identify a narrow list of ASVs that are associated with either high or low yield. There was a total of 56 ASVs found in the trunk compartments (Fig. 7A) and 120 ASVs in the rhizo-compartments (Fig. 7B). Random forest models were used to rank these ASVs and identify the ten most important for predicting yield in both the trunk and rhizo-compartments. For the trunk compartment model, LASSO regression identified seven of the top ten most important ASVs, Indicator Species identified four, Spearman correlation three, and Differential Abundance two. While in the rhizo-compartment model, Indicator Species identified the most with five, followed by LASSO regression with four, and both Differential Abundance and Spearman correlation with two.

Multiple linear regression models were used to quantify the relative contributions to yield for each of the ten highest impact ASVs while controlling for the cultivar effect. The model utilizing trunk compartment ASVs was able to explain 51% of the total yield variance (Fig. 7C) while the rhizo-compartment ASV model was able to explain 60% of the total yield variance (Fig. 7D). In the trunk compartment model, cultivar explained 7.4% of yield variance while in the rhizo-compartment model, it explained 7.6% of yield variance. The remaining 43.4% and 52.6% of yield variance was explained by the ten ASVs in each model.

To evaluate the models’ generalizability and check for overfitting, a post hoc analysis was conducted, where the models were trained on 80% of the data and tested on the remaining 20%. Both models demonstrated moderate generalizability and lacked overfitting, with the rhizo-compartment model performing better, as indicated by low differences in root mean squared error (RMSE) between training and test data, and test R-squared values of 0.33 for the trunk compartment model and 0.73 for the rhizo-compartment model. To further validate the models, other dependent variables associated with plant vigor were used to test if the high-impact ASVs remained predictive. Trunk diameter and cane weight as dependent variables were tested when the data was available across the three years of the study. Both trunk- and rhizo-compartments ASV abundance was highly predictive of these alternate measures of plant vigor across the different years tested, supporting their role as true contributors to general plant growth (Supplemental Fig. 6). The only exception was a near-zero R-squared value for rhizo-compartment ASVs predicting trunk diameter in the first few months after planting.

Of the ten high-impact ASVs identified in the trunk compartment model, only four contributed positively (Table 2; *Clonostachys*, *Pantoea*, *Serratia*, *Erwinia*) and six negatively (Table 2; *Couchioplanes*, *Blastococcus*, *Nocardioides*, *Phaeoacremonium*, *Nocardia*, *Micromonospora*) to yield. In contrast, of the ten high impact ASVs identified in the rhizo-compartment model, eight contributed positively (Table 2; *Flavobacterium*, *Rurimicrobium*, *Devosia*, *Klebsiella*, *Franconibacter*, *Rhizophagus*, *Humicola*, *Serratia*) and two negatively (Table 2; *Novosphingobium*, *Hypocreales*) to yield. *Serratia* was the only taxa that contributed positively to yield in both above and belowground compartments. In the trunk compartment model, the total contribution to yield variance of the positively related ASVs was 15.8% and 27.5% for the negatively related ASVs. In the rhizo-compartment model the total contribution to yield variance of the positively related ASVs was 43.2% and 8.8% for the negatively related ASVs. All but three ASVs listed in Table 2 (*Franconibacter*, *Rhizophagus*, *Flavobacterium*) originated from the nurseries (Supplemental Fig. 7).

**Table 2:**
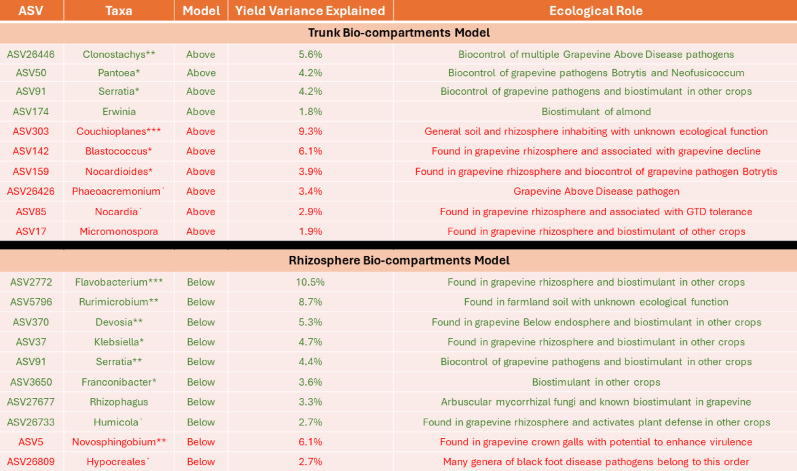
Relative contribution to yield of the ASVs from the trunk and belowground predictive models and the ecological role of their associated taxonomic group

The statistical significance of individual variables is indicated by the following *P*-values: ≤ 0.001 (***), ≤ 0.01 (**), ≤ 0.05 (*), and ≤ 0.1 (`). Green indicates a positive and red a negative contribution to yield. Proportion of total yield variance explained for each variable was calculated using the Lindeman, Merenda, and Gold (LMG) method.

## Discussion

This study establishes that nursery plant materials have a lasting imprint on the endospheric microbiome associated with young grapevines and possibly influence vigor and productivity of vineyards. It provides the evidence that infection with vascular pathogens inherited from nurseries does not necessarily equate to vine death post-planting. We use machine learning techniques to determine whether microbe abundance data from the trunk and below ground microbiome has any predictive power for yield outcomes. To our knowledge this research represents the first attempt to implement quantitative modelling of yield in a perennial crop based on microbial data. This has resulted in the identification of high-impact microbes that warrant further research for their role in grapevine yield.

Previous studies have identified the vineyard soil as a major influence and source of the epiphytic microbiome of above ground tissues and especially the carposphere [10]. As a result, the vineyard native microbiome plays an active role in shaping wine characteristics and has been recognized as an important component of the terroir. However, our data suggest that this paradigm does not necessarily apply to communities living inside the vine where the nursery origin is a major driver of the microbial identity, at least in the initial lifespan of the vineyard. Cultural practices implemented in nurseries have been shown to be determinants of the microbial communities inhabiting woody tissues of grafted vines [18]. In our study, vines from both nurseries displayed distinct microbiomes that converged overtime following planting in the vineyard, although the influence of the nurseries on community composition was still significant after three years. Our data indicated that 15% and 12% of the fungal and bacterial ASVs across the trunk and rhizo-compartments of three-year-old grapevines originated from the nurseries. The grapevine also acquired about 20% of its core microbiome from the vineyard within a year of planting and these shifts in community composition continued each year thereafter. Interestingly, the post-planting microbial turnover occurred at different rates in the rhizo- and trunk compartments for bacteria and fungi. The rhizosphere and root communities were highly dynamic with a high turnover of many taxa each year. This is likely reflective of the host active recruitment process of autochthonous soil microbes to adapt to its new environment [28]. In contrast, the trunk endosphere was relatively stable, especially for fungal communities, and underwent a slow turnover process perhaps because it is a less hospitable environment (i.e., low nutrient availability, negative pressure of the xylem) and due to the restricted movement of microbes from the roots to the internal aboveground tissues [10]. Together, his suggests that while the grapevine microbiome from the nursery persists over time, it is being replaced with the acquisition of native microbes from the vineyard, with a faster turnover in rhizo-compartments than in the trunk. The bacteriome also appears to exhibit a higher level of plasticity than the mycobiome.

Pathogenic fungi inhabiting the trunk were important members of the core community inherited from nursery vines (e.g., *Fusarium*, *Phaeoacremonium*). Infection with GTD-pathogens comes from infected mother vine cuttings and from contaminated water and soil substrates used during propagation [15, 29]. The vine-associated pathobiome clearly displayed a distinct profile at 3 years post-planting compared to its initial nursery composition, with infection of new soilborne pathogens from the vineyard (e.g., *Ilyonectria*, *Truncatella*), and an increase in incidence and abundance of latent nursery pathogens associated with young vine decline (e.g., *Phaeoacremonium*, *Phaeomoniella*) and black foot (e.g., *Dactylonectria*, *Rhizoctonia*), while others faded away (e.g., *Pleurostoma*, *Neonectria*) or vanished (e.g., *Diplodia*, *Diaporthe*). Yet only a fraction of the vines died in the first year following planting, which is common for new vineyards. No vines were lost in subsequent years nor did they expressed typical disease symptoms (i.e., root decay, wood dieback) despite the high infection level with GTD-pathogens, indicating that their presence does not guarantee significant vine decline or death. There is building evidence that vine stress (e.g., drought, extreme heat) is required for these opportunistic organisms to become pathogenic [16] and that those conditions were not met in our experimental vineyard. However, some of these organisms (i.e., *Phaeoacremonium*) appear to be linked to a moderate decrease in vine productivity.

A statistical model was used to identify in both the rhizo- and trunk compartments the ten most impactful microbial ASVs within the microbiome community that could predict yield. The deployment of machine learning algorithms for microbiome-based models have become powerful tools to predict yield levels [30, 31] and risks of soilborne diseases [32, 33] in several cropping systems and in many cases provide equal or stronger predictions than soil models that are based on physicochemical properties [30, 32]. Although our trunk and belowground models could predict 51% and 60% of yield variance in young vineyards, it does not necessarily mean that the identified microbes directly affected fruit production. Yield is complex and multifactorial by nature and confounding variables such as viticultural practices, edaphic and environmental factors, that drive fruit set and microbiome structure could have impacted our analysis, as shown in other systems [34–37]. The limited sample size, lack of soil nutrient analysis and acquisition of weather data in this study has certainly compounded these issues [32]. Large-scale sampling from vineyards across viticulture areas coupled with input of vineyard metadata should be incorporated in future studies to generate more robust predictions.

Our data indicated that there was no signature of high or low yielding vines at the microbial community level, but rather that a small group of specific microbes were the main drivers of these differences. Interestingly, of the nineteen unique high impact ASVs identified in the vineyard, sixteen originated from the nursery, reinforcing the importance of the nursery-inherited microbiome on vineyard health. Most of the ASVs from the trunk were found to be associated with low yielding vines, whereas only two were identified in the belowground tissues. Those included several taxa involved with young vine decline in vineyards, namely *Phaeoacremonium* one causal agent of Petri disease [15, 38, 39], *Hypocreales* a group of fungi associated with black foot [40, 41] and *Novosphingobium* known to enhance virulence of *Allorhizobium vitis* the causal agent of crown gall [40, 41]. Again, none of the vines expressed typical symptoms of those diseases (i.e., wood dieback or gall formation), but these inhabiting pathogens likely took resources away from the host and reduced vigor and fruit set. In addition, *Actinobacteria* were strong indicator of low yielding vines including *Couchioplanes*, *Blastococcus*, *Nocardioides*, *Nocardia*, and *Micromonospora*. *Actinobacteria* are commonly found in soil and plant rhizosphere ecosystems [42–45] and are known to produce a wide range of biologically active compounds that provide several benefits to the host including biological control, acquisition of nutrients and plant growth promoting capabilities [44, 46, 47]. However, their biological function in grapevine is unclear. Their association with low yielding vines may indicate that under specific conditions those taxa could become harmful perhaps because of competition with the host for nutrient acquisition or the production of compounds that are phytotoxic to grapevine. Alternatively, stressed vines might actively recruit these microbes in a stress-mitigation response that is ultimately unsuccessful. The majority (10 of the 12 ASVs) of the beneficial organisms associated with high yielding vines originated from the nursery and encompassed known biological control agents of several grapevine pathogens, including those causing GTD such as *Clonostachys* [48], *Pantoea* [49, 50], *Serratia* [51, 52], and *Rhizophagus* [53–55]. Perhaps the persistence of these taxa post-planting promotes vine resilience by antagonizing GTD pathogens or countering their negative impact. These organisms are also reported to provide benefits to the host in other plant systems, including promoting plant growth and protection against biotic and abiotic stresses [56–59].

## Conclusion

In summary, this research highlights the importance of the grapevine life stage in nursery on vineyard outcomes. Nursery vines carry both beneficial and pathogenic organisms that can sway the performance of young vineyards. It also adds another dimension to the definition of terroir, in the sense that microbes are not only of vineyard origin and foreign microbes from nurseries can influence vineyard success and perhaps wine making processes. Our work also underscores the potential to improve nursery and vineyard management practices through microbiome-based strategies. One application of this research is to develop a decision-making platform for growers by coupling the microbial fingerprint of grapevine with environmental metadata (soil chemistry, weather data) from vineyards to build robust plant health and yield predictions. Another applied aspect of this work lies in the discovery of biological inoculants for engineering the plant microbiome in nurseries to enhance resiliency and productivity of vineyards.

## Supplementary Information

Below is the link to the electronic supplementary material.

Supplementary Material 1

## Data Availability

Sequence data were deposited into the NCBI database under BioProject accession number PRJNA1370506; ncbi.nlm.nih.gov/bioproject/PRJNA1370506.
