# Supplementary material for: Microbial succession from nursery to vineyard highlights the role of beneficial and pathogenic microbes in young vineyard yield
Source: Environ Microbiome. 2026 May 4;21:81. doi: 10.1186/s40793-026-00905-8 (PMC13312510; doi:10.1186/s40793-026-00905-8)
Supplement: Supplementary file 1 — Supplementary Material 1 [file 40793_2026_905_MOESM1_ESM.docx]

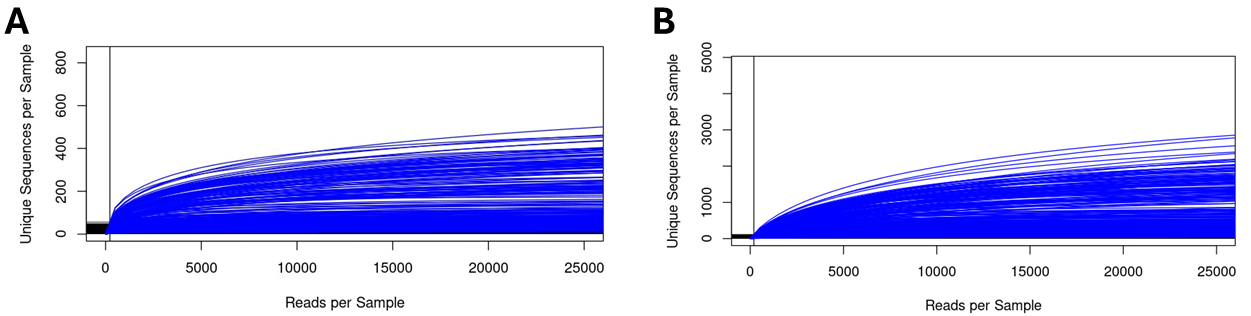


**Supplemental Figure 1.** Rarefaction Analysis for (A) Fungi and (B) Bacteria. Based on analysis, rarefaction of 10,000 reads was deemed acceptable.


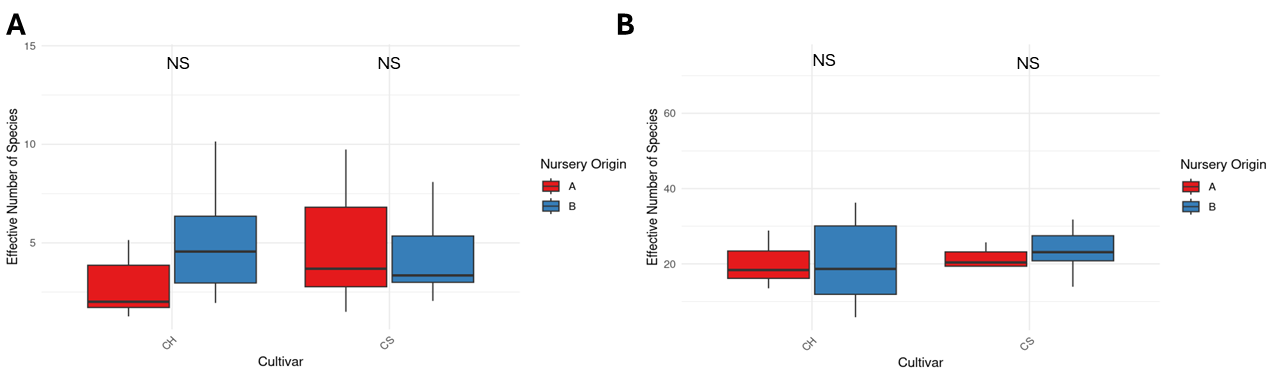


**Supplemental Figure 2.** Boxplots showing no significant differences (Kruskal-Wallis test, *P* > 0.05) in the effective number of species index for fungal (A) and bacterial (B) communities between the two nurseries for the cultivars Chardonnay (CH) and Cabernet Sauvignon (CS).


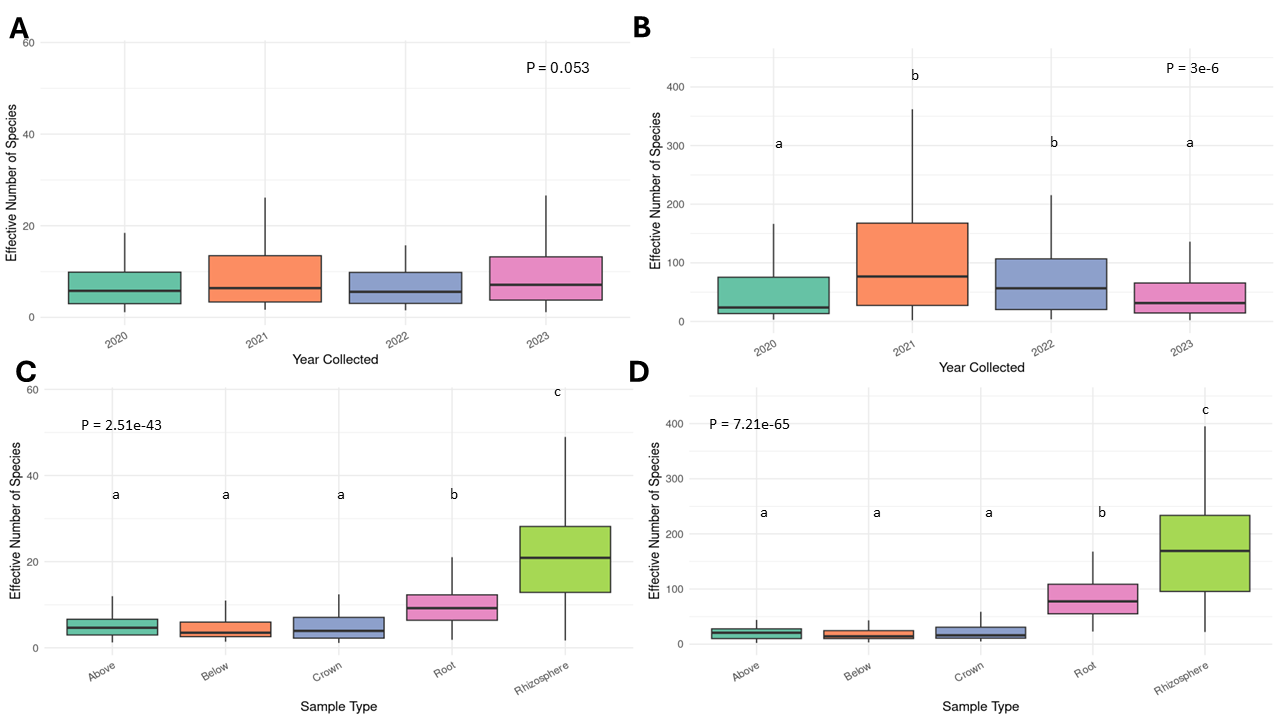


**Supplemental Figure 3.** Boxplots showing the effective number of species for fungal (A) and bacterial (B) communities collected from grapevine plant material across four years (2020–2023). Both fungal and bacterial alpha diversity varied significantly by year (Kruskal–Wallis test; fungi *P* < 0.01, bacteria *P* < 0.001). Letters indicate statistically distinct groups based on pairwise post-hoc comparisons (*P* < 0.05). The effective number of species index for fungal (C) and bacterial (D) communities across plant compartments (Above, Below, Crown, Root, Rhizosphere). Both datasets showed significant differences in effective number of species among compartments (Kruskal–Wallis test; fungi *P* < 0.001, bacteria *P* < 0.001), with the rhizosphere exhibiting the highest species richness.


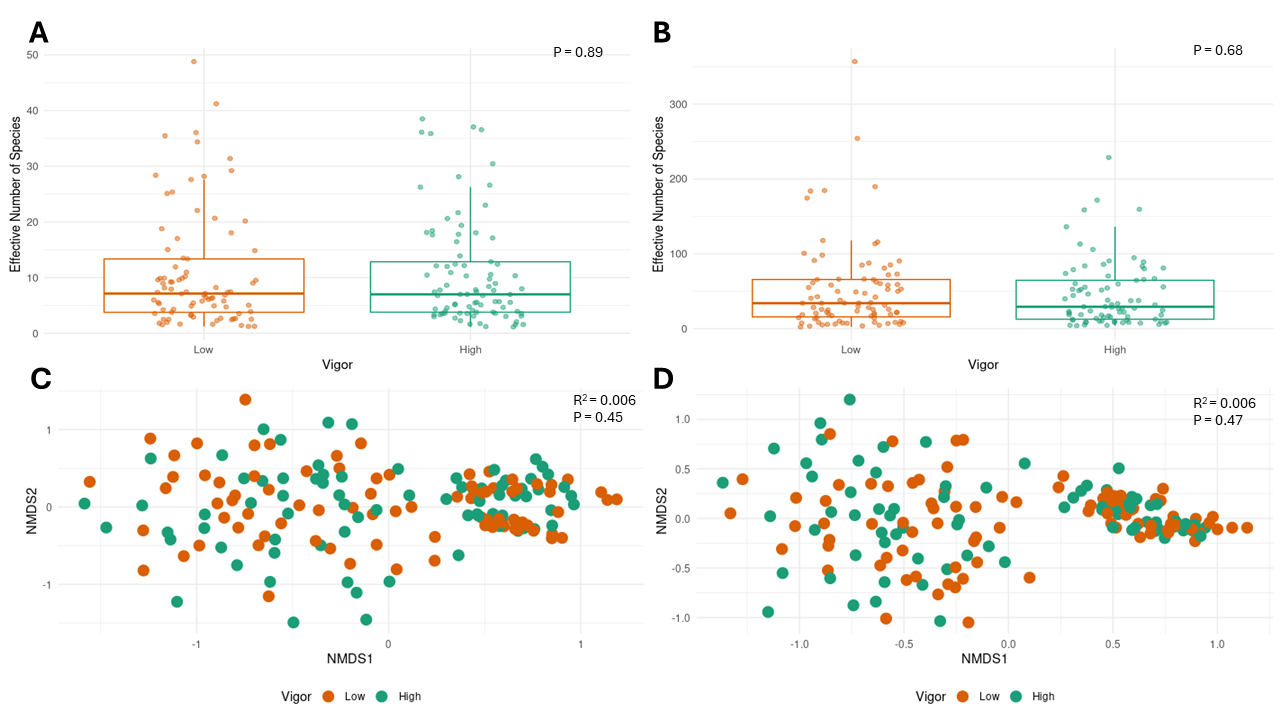


**Supplemental Figure 4.** Boxplots showing effective number of species index for fungal (A) and bacterial (B) communities associated with low- and high-vigor vines. No significant differences in alpha diversity were observed between vigor categories (Wilcoxon signed-rank test; fungi *P* > 0.5, bacteria *P* > 0.5). Non-metric multidimensional scaling (NMDS) ordinations based on Bray–Curtis dissimilarities depicting fungal (C) and bacterial (D) community composition between low- and high-vigor vines. Beta diversity did not differ significantly between vigor groups (PERMANOVA; fungi R² = 0.006, *P* > 0.5; bacteria R² = 0.006, *P* > 0.5).


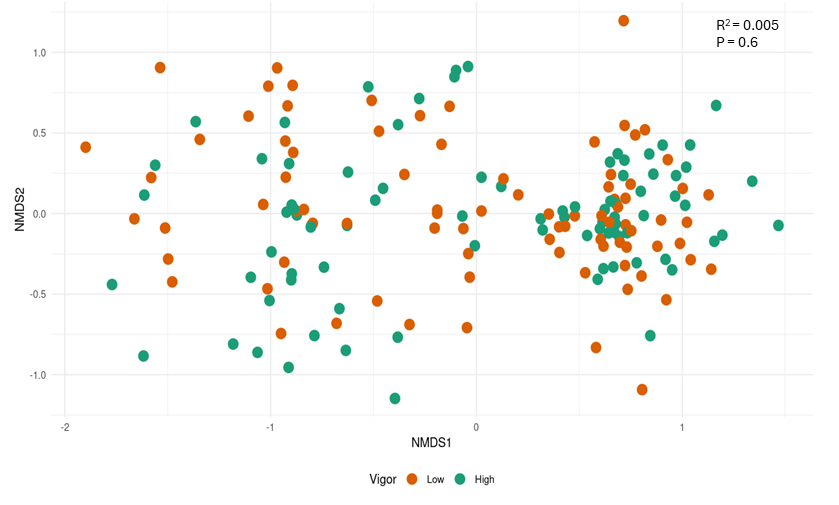


**Supplemental Figure 5.** Non-metric multidimensional scaling (NMDS) ordination based on Bray–Curtis dissimilarities depicting variation in the composition of grapevine-associated pathogenic fungal genera between low- and high-vigor vines. Community composition did not differ significantly between vigor groups (PERMANOVA, R² = 0.005, *P* > 0.5).


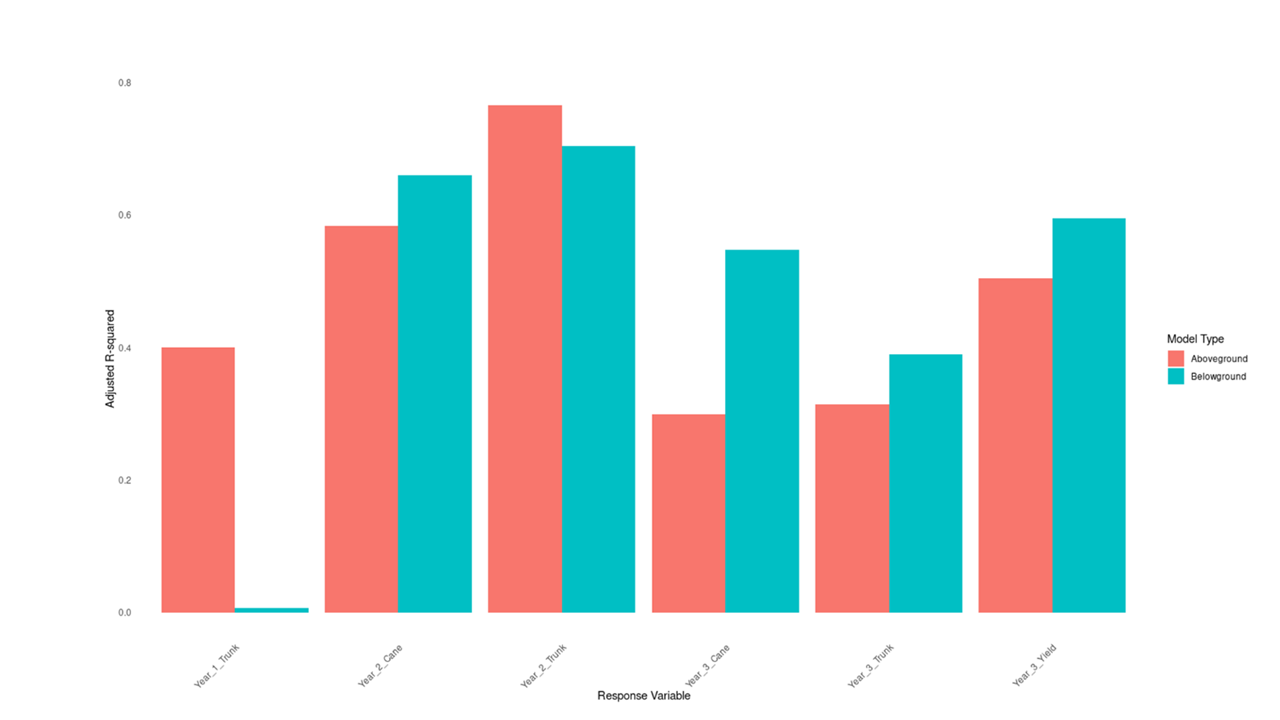


**Supplemental Figure 6.** The high impact ASVs identified in the Year 3 Yield Models are also predictive of other measures of vigor over three years, indicating their generalizability. The adjusted R-squared values of linear regression models predicting various measures of vine vigor in different years (trunk diameter and cane weight) were similar and in some cases higher than the adjusted R-squared values of models trained on Year 3 Yield. Generally, the rhizo-compartment (belowground) models outperformed the trunk compartment models (above ground), except in the cases of year 1 and 2 trunk diameter.

**
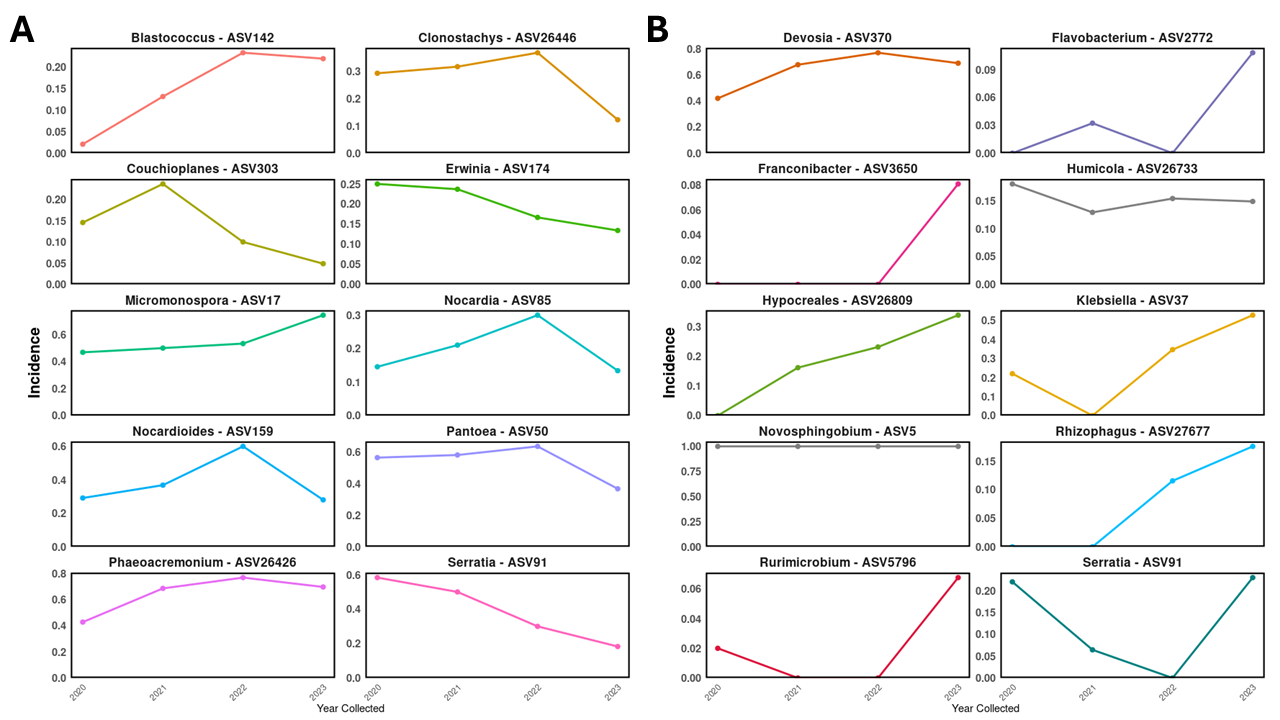
**

**Supplemental Figure 7.** All high impact trunk compartment ASVs (A) originate from the nursery and a majority of high impact rhizo-compartments ASVs (B) originate from the nursery. All but three ASVs *Franconibacter*, *Rhizophagus*, and Flavobacterium originated from the nurseries. Samples were filtered to remove instances where high impact ASVs were below 0.1% abundance before calculating the incidence of each ASV over the four years during the nursery to vineyard transition.
